# Law-suits upon Shares

**Published:** 1867-11

**Authors:** 


### Law-suits upon Shares.

We take pleasure in publishing the testimony in the case reported in this Journal, brought against Dr. Merrill E. Shaw, for having caused the death of a patient by the subcutaneous administration of a grain of morphine. The verdict or rather the refusal of Judge Clinton to submit the case to the deliberation of the jury is doubly satisfactory. We have learned since the trial, that there were one or two men upon the jury ready to find handsomely for the widow, had opportunity been granted, showing, if so, that our rights and liberties are no more safe in the hands of a jury, than was the life of the lamented defendant in the hands of Western savages.

Within the last few years an unsafe and unprofessional habit has obtained countenance, by which members of the legal profession obtain the conduct of suits upon shares. The terms are such that the chief operator and instigator loses nothing if unsuccessful, and gains one-half or even more, if he wins. The plaintiff advances the necessary costs of the suit, and the operator furnishes the advice and works up the case. But for this custom, most of the unjustifiable suits now brought before the courts would never be entered upon the records, and many, very many, who now appear as plaintiffs would never have mistrusted themselves aggrieved. We do not think that this operates against medical men more frequently than against all others; it may be seen everywhere, and every fair-minded individual can see the natural effect of such practice.

We have plenty to do to expose and correct the unprofessional habits of members of our own profession, without making attack upon any other, and it is quite provoked or we should not now undertake to condemn a practice which has shown itself in so many instances, as to make further silence impossible. This case against Dr. Shaw was pressed, after telegraphic report of the death of the defendant; and even delay in the trial refused. The whole legal procedure was based upon nothing, and carried on upon the expectation that a jury might favor a widow regardless of right, and herself and *friends* be indirectly benefited by the natural sympathy which men feel for women.

